# Socioeconomic inequality and decomposition of core capacity in global health security: the role of health system

**DOI:** 10.7189/jogh.15.04234

**Published:** 2025-10-24

**Authors:** Minmin Wang, Mengze Liu, Zuokun Liu, Hui Yin, Zhen Xu, Minghui Ren

**Affiliations:** 1 China Center for Health Development Studies, Peking University, Beijing, China; 2 Department of Global Health, School of Public Health, Peking University, Beijing, China; 3 Health Emergency Center, Chinese Center for Disease Control and Prevention, Beijing, China; 4 Institute for Global Health, Peking University, Beijing, China

## Abstract

**Background:**

Core capacities in preparedness, detection, and response to health emergencies were fundamental to achieving health equity. However, evidence on the socioeconomic inequalities in country-level global health security (GHS) capacities remains limited, and the role of the health system in contributing to these disparities is insufficiently understood.

**Methods:**

We assessed the socioeconomic inequalities in country-level GHS capacities and decomposed the role of health systems in shaping these inequalities by applying the GHS index and Joint External Evaluation. We conducted the decomposition based on a linear regression model with the determinants reflecting economic status, social development, and the health system. We also decomposed the changes in the concentration index (CI) using an Oaxaca-type decomposition.

**Results:**

Disparities in country-level health security capacities in response to health emergencies have been reported (CI = 0.143; *P* < 0.001). Economic status accounted for 44.639% of the total inequality, while social development accounted for −13.386%, and the health system for 67.454%. Disparity of the health system was the leading cause of inequality in health security capacity. From 2019 to 2021, economic status contributed to the change of inequality by 70.168%, social development by −62.544%, and the health system by −42.219%.

**Conclusions:**

Our results emphasised the fundamental role of health system strengthening in improving health security. Strengthening health systems, particularly through the enhancement of the universal health coverage framework by recognising the interconnection between health systems and health security, offers a viable strategy for achieving equality-driven GHS goals.

The COVID-19 pandemic significantly heightened global awareness of the persistent threat posed by emerging infectious diseases, thereby positioning global health security (GHS) as a pivotal concern on the international health agenda. In 2024, the Seventy-seventh World Health Assembly reached a landmark agreement on a series of wide-ranging amendments to the International Health Regulations (IHR) [1]. These amendments aim to strengthen global capacities for pandemic preparedness and response, with a distinct focus on equity. In parallel, continued negotiations on a global pandemic agreement underscore the importance of improving international collaboration, coordination, and equity in efforts to prevent, prepare for, and respond to future pandemics.

Global disparities in response capabilities during infectious disease outbreaks have long been a source of concern [2]. During the 2014–15 West African Ebola epidemic, inadequate diagnostic systems and inequitable access to reliable testing resulted in considerable delays at every stage of the outbreak response [3]. Experience in the COVID-19 pandemic revealed inequalities in access to vaccines, therapeutics, and essential healthcare, which contributed to wide variations in COVID-19 mortality rates and excess all-cause mortality [4–6]. These inequalities have driven a growing consensus around the need for global health frameworks that prioritise equality and equity, particularly for low- and middle-income countries. As a response, the 2024 IHR amendments explicitly incorporated ‘promote equity and solidarity’ as core principles. The draft international instrument on pandemic prevention, preparedness, and response positions equity as a fundamental goal, striving to eliminate ‘unfair, avoidable, or remediable differences among and between individuals, communities, and countries’. Strengthened core capacities in response to the health emergencies were central to operationalising these principles. The IHR amendments planned to establish a Coordinating Financial Mechanism to support identification of, and access to, financing required to ‘equitably address the needs and priorities of developing countries, including for developing, strengthening and maintaining core capacities’, and other pandemic emergency prevention, preparedness, and response-related capacities. Requirements for key functions such as prevention, surveillance, reporting, notification, verification, preparedness, and response, as well as international collaboration through mechanisms involving designated airports, ports, and ground crossings, have been updated in the IHR amendments.

Resilient health systems and universal health coverage (UHC) are critical determinants of countries’ capacities to ensure GHS. The COVID-19 pandemic revealed that no country could effectively detect, assess, notify, and report health security threats or respond promptly and effectively without first ensuring universal access to quality healthcare services [7]. The Lancet Commission on the synergies between UHC, health security, and health promotion emphasised that while GHS focusses on developing public health core capacities, it overlooked essential primary healthcare functions such as curative services, patient management, and the ability to handle clinical surges [8,9]. The COVID-19 pandemic tested national health systems' capacities to withstand health shocks while maintaining routine healthcare functions, uncovering the need for health system resilience. This realisation underscored that health crises did not occur in a vacuum; they were deeply influenced by the structural capacities of national health systems, including financing, service coverage, and a well-trained workforce [10].

Health emergencies exposed the disparities in GHS core capacities across countries and provided a critical opportunity to explore pathways for addressing these inequities through targeted health system strengthening. However, evidence on the socioeconomic inequalities in country-level GHS capacities remains limited, and the role of the health system in contributing to these disparities is insufficiently understood. Therefore, we aim to assess the socioeconomic inequalities in country-level GHS capacities and to decompose the role of health systems in shaping these inequalities, with the results providing quantitative evidence and informing strategies to strengthen equality-oriented GHS.

## METHODS

### Data source

We retrieved two sets of the indicators – GHS index (GHSI) [11] and Joint External Evaluation (JEE) – to evaluate the country-level core capacity for pandemic emergency prevention, preparedness and response.

The GHSI framework incorporates feedback from an International Panel of Experts and lessons from the COVID-19 pandemic and other epidemics and pandemics. The GHSI is assessed through six categories (*i.e.* prevention, detection and reporting, rapid response, health system, compliance with international norms, and risk environment), 37 indicators, 96 sub-indicators, and 171 questions. A team of more than 80 experienced field-based researchers collects publicly available data to evaluate the country-level score. The first two editions of the GHSI were published in 2019 and 2021. Detailed methodology of the GHSI was listed on its website [12]. Each category is scored on a 0–100 scale, and the overall score (0–100) for each country is a weighted sum of the scores in the six categories. Each category is standardised based on the sum of its underlying indicators and sub-indicators, and a weight is assigned to each. To facilitate reproducible comparisons across countries, categories and indicators, measurements have been normalised to a scale of 0–100. We retrieved and used the 2019 and 2021 versions of the GHSI data.

The JEE is a multisectoral process and consists of a country self-assessment on the IHR, which is shared with an external team of the World Health Organization (WHO) and non-WHO experts, who then visit and make a week-long independent assessment [13,14]. The JEE estimates the country-level capacity by the IHR requirement, regarding prevention, detection, response, and IHR-related hazards and points of entry [15]. We calculated a geometric mean of the JEE indicators as the total score reflecting countries’ preparedness in response to the health emergencies.

### Aims and outcomes

We aim to assess the socioeconomic inequalities in country-level GHS capacities and to decompose the role of health systems in shaping these inequalities.

### Primary outcome

Our primary outcome was the percentage contribution of economic status, social development, and the health system to the inequality of the GHS capacity.

### Secondary outcome

Our secondary outcome was the contribution of economic status, social development, and the health system to changes in GHS capacity inequality.

### Determinants of health security capacity

Following the conceptual framework of the health security assessment [16], the economic status, social development, and health system were the key pillars influencing countries’ core capacity. To balance the number of countries included in the analysis due to data availability and the completeness of the information, we applied two sets of the determinants of health security capacity (Table S1 in the **Online Supplementary Document**), both representing economic status through gross national income (GNI) per capita, social development through the population, and world governance through the world governance index. Regarding the health system, the first set utilised UHC as its primary focus, with health financing as a key component. The second set expanded this framework to include the health workforce and health supplies. We also conducted an association analysis between the socioeconomic determinants and health security capacity (Table S2 in the **Online Supplementary Document**).

### Statistical analysis

We measured and explained the socioeconomic inequality in the health security capacity using the concentration index (CI) and decomposition method [17]. We estimated the CI as follows:



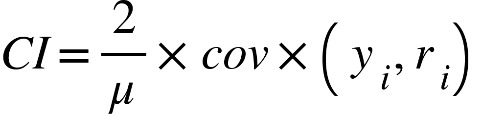



Where *y_i_* is GHI index for country *i*, *r_i_* is the fractional ranking of countries according to wealth index and μ is the mean of *y_i_*. The value of this index falls between −1 and 1. A negative CI indicates higher health security capacity among the poor (pro-poor) while a positive value suggests greater health security capacity among the rich (pro-rich). The higher the absolute value of the CI is, the greater the extent of inequality. We estimated the CI regarding the GHS and its six categories using the data from 2019 and 2021 separately.

We conducted the decomposition of socioeconomic inequality of the country-level health security index according to the World Bank’s suggestion [18]. To identify the relative contribution of various factors to the socioeconomic-related inequality in barriers to accessing healthcare, we performed a decomposition of the CI. For any linear additive regression model of health security capacity (*y*):



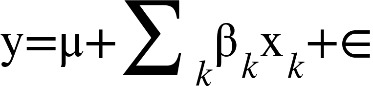



The CI for *y* is given as:



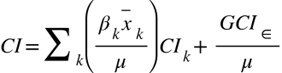



Where *y* is the health outcome variable (in this case socioeconomic-related inequality of health security capacity), *x_k_* is a set of determinants of the health outcome, α is the intercept, β*_k_* is the coefficient of *x_k_*, *μ* is the mean of *y*, x̄_k_ is the mean of *x_k_*, CI*_k_* is the CI for *x_k_*, GCI_∈_ is the generalised CI for the error term (∈), is the elasticity of y with respect to x̄_k_ [19]. The GCI_∈_ captures the inequality associated with the residual (error term) – *i.e.* the part of the inequality not explained by the observed covariates. The elasticity measures the strength of the association between a determinant and the health security index. A larger absolute elasticity means a stronger influence on the outcome. The component contribution is the product of elasticity and the determinant’s CI, showing how much each factor contributes to the total inequality.

Furthermore, we also decomposed the changes in the CI according to the Oaxaca-type decomposition [20]. This method, initially developed for analysing wage differentials, has been adapted in health economics to disentangle the sources of changes in income-related health inequality. The change in the CI can be approximated by the following:



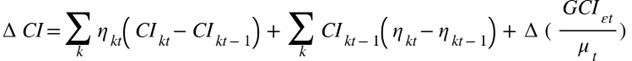





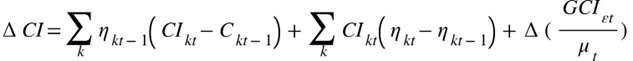



Where *t* indicates time period and ∆ denotes first differences, specifically, it enables the decomposition of the overall change in the CI into two distinct components: changes in the distribution (or inequality) of the determinants of health across income groups, and changes in the responsiveness (*i.e.* elasticities) of health outcomes to these determinants. By separating compositional effects from structural effects, the Oaxaca-type decomposition provides nuanced insights into the mechanisms driving temporal variations in socioeconomic health disparities. We conducted all statistical analyses using STATA, version 14.0 (Stata Corp LLC, Texas, USA). All tests were two-sided, and we considered *P*-values <0.05 as statistically significant.

## RESULTS

### Average level and inequality of GHSI

We evaluated a total of 195 State Parties to the IHR 2005, and assessed their preparedness gaps in the public health capacity. The mean (x̄) of the overall GHSI reached 38.91 (95% confidence interval = 36.98–40.83) in 2021; one country was placed in the top tier of the GHSI, and over 60% of the State Parties were listed in the bottom two tiers (Table S3 in the **Online Supplementary Document**). Among the six categories, the average score of prevention capacity was the lowest (x̄ = 28.45; 95% confidence interval = 25.93–30.97), and over 40% of the State Parties were listed in the bottom tier. The security capacity regarding risk environment dimension received the highest score (x̄ = 55.84; 95% confidence interval = 53.74–57.94) and 12 State Parties reached the top tier.

**Figure 1:**
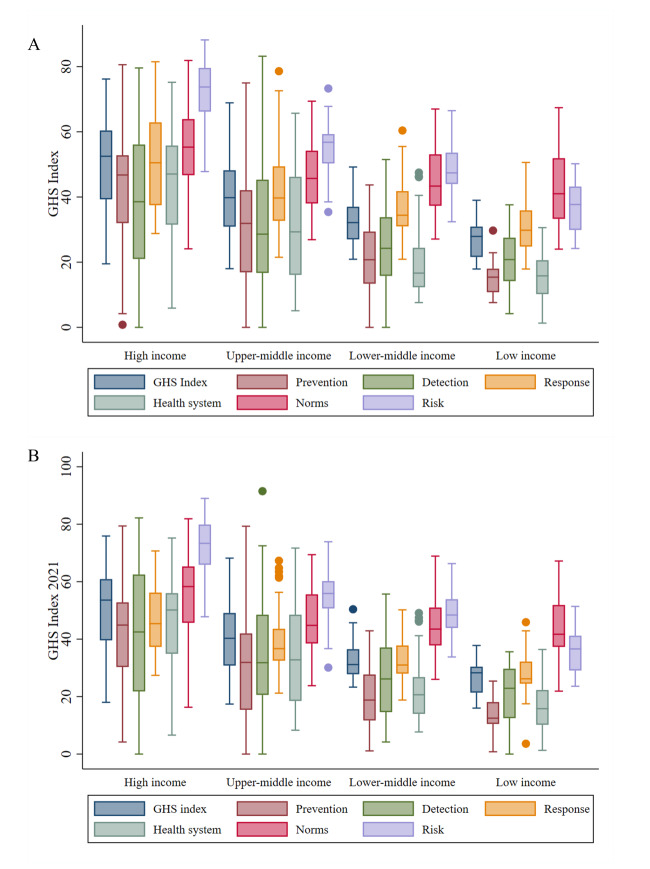
Average level of the GHSI, among the total score and its six categories, by income groups. **Panel A.** Average level of the GHSI, among the total score and its six categories in 2021, by income groups. **Panel B.** Average level of the GHSI, among the total score and its six categories in 2019, by income groups. GHSI – global health security index.

Score of GHSI varied across the country-level socioeconomic strata. The average GHS score was the highest for high-income (x̄ = 50.69; 95% confidence interval = 47.17–54.21), followed by upper-middle income (x̄ = 39.86; 95% confidence interval = 36.57–43.14), and the lowest for low-income countries (x̄ = 26.40; 95% confidence interval = 23.88–28.92). The score for the detailed six GHS categories displayed a similar pattern (**Figure 1**; Table S3 in the **Online Supplementary**). We calculated the CI to measure the inequality of health security capacity in terms of the economic level (using GNI per capita as the agent variable) (Table S4 in the **Online Supplementary Document**). The CI of the overall GHS score reached 0.142 (*P* < 0.001). Among the six GHS categories, the prevention dimension was of the highest disparity of 0.248 (*P* < 0.001), and compliance with international norms was of the smallest inequality of 0.057 (*P* < 0.001). Considering the income levels, countries in the upper-middle income levels were of the highest disparity, with a CI of 0.093 (*P* < 0.001), and countries in the low-income levels were of the lowest disparity, with a CI of 0.023 (*P* < 0.001).

### Decomposition of the GHS inequality

**Figure 2:**
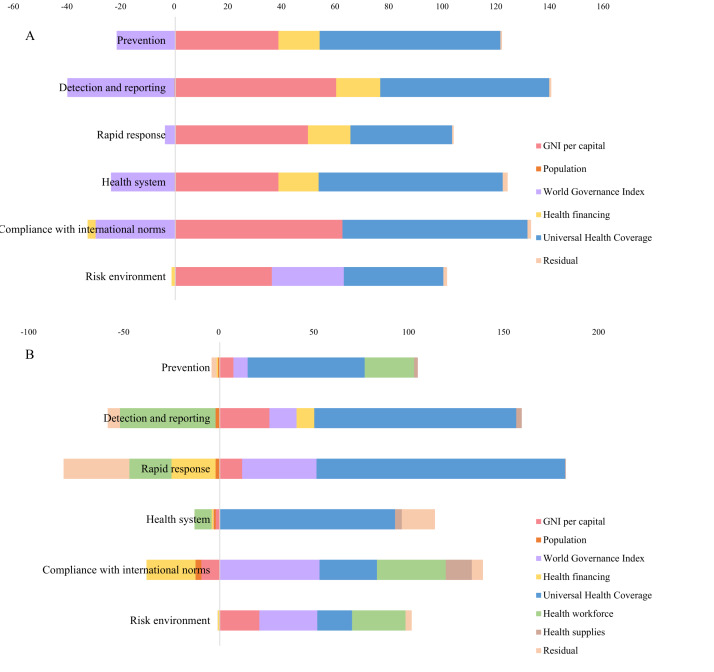
Percentage contribution of economy, social development, and health system in shaping the socioeconomic inequality of health security capacities, by the six categories. **Panel A.** Decomposition applied the determinants framework 1. **Panel B.** Decomposition applied the determinants framework 2. GNI – Gross National Income.

Based on the framework of determinants of health security capacity, the inequality of health security capacity was decomposed into three dimensions: economic status, social development, and health system. In the first set of the determinant’s framework, a total of 181 countries with available covariate data were included in the analysis (**Table 1**). Economic status accounted for 44.639%, social development for −13.386%, and health system for 67.454% of the total inequality. Disparity of the health system was the leading cause of inequality in health security capacity. Decomposition using the second set of determinants framework also suggested similar results (38 countries included), with economic status accounting for 10.293%, social development for 18.041%, and health system for 72.425% of the total inequality. Among the health system, health service coverage was the dominant factor explaining the health security disparity, with a CI of 0.126 and contributing to 56.991% of the total disparity. We conducted a similar decomposition in terms of six GHSI categories (**Figure 2**; Tables S5 and S6 in the **Online Supplementary Document**). Health system, especially the service delivery and coverage, remained the dominant explainer of prevention (82.722%), detection and reporting (79.439%), rapid response (53.764%), and health system (83.700%), compliance with international norms (65.992%), and risk environment (35.839%).

**Table 1:** Decomposition of socioeconomic inequality in health security capacities (2019, 2021, and the change between the two years)

	2021				2019				Change	
	**Elasticity**	**CI**	**Contribution**	**% contribution**	**Elasticity**	**CI**	**Contribution**	**% contribution**	**Contribution**	**% contribution**
**Framework 1**										
GNI per capita	0.126	0.505	0.064	44.639	0.134	0.503	0.067	49.337	0.0044	70.168
Population	0.009	−0.023	0.000	−0.143	0.010	−0.044	0.000	−0.324	0.0002	2.762
World Governance Index	−0.147	0.128	−0.019	−13.242	−0.176	0.125	−0.022	−16.141	−0.0041	−65.306
Health financing	0.087	0.172	0.015	10.462	0.073	0.172	0.013	9.231	−0.0023	−37.449
UHC	0.647	0.126	0.081	56.991	0.646	0.126	0.081	59.739	−0.0003	−4.770
Residual			0.002	1.294			−0.003	−1.842		
**Framework 2**										
GNI per capita	0.029	0.505	0.015	10.293	0.113	0.503	0.057	41.574	0.0420	679.732
Population	0.083	−0.023	−0.002	−1.327	0.009	−0.044	0.000	−0.291	0.0030	55.902
World Governance Index	0.215	0.128	0.028	19.368	0.009	0.125	0.001	0.857	−0.0260	−413.174
Health financing	−0.033	0.172	−0.006	−3.943	0.064	0.172	0.011	8.084	0.0170	266.674
UHC	0.815	0.126	0.102	71.866	0.594	0.126	0.075	54.904	−0.0280	−450.970
Health workforce	0.007	0.343	0.003	1.768	0.037	0.337	0.013	9.258	0.0100	166.172
Health supplies	0.023	0.169	0.004	2.734	0.009	0.191	0.002	1.317	−0.0030	−45.152
Residual			−0.001	−0.759			−0.021	−15.703		

CI – concentration index, GNI – Gross National Income, UHC – universal health coverage

**Figure 3:**
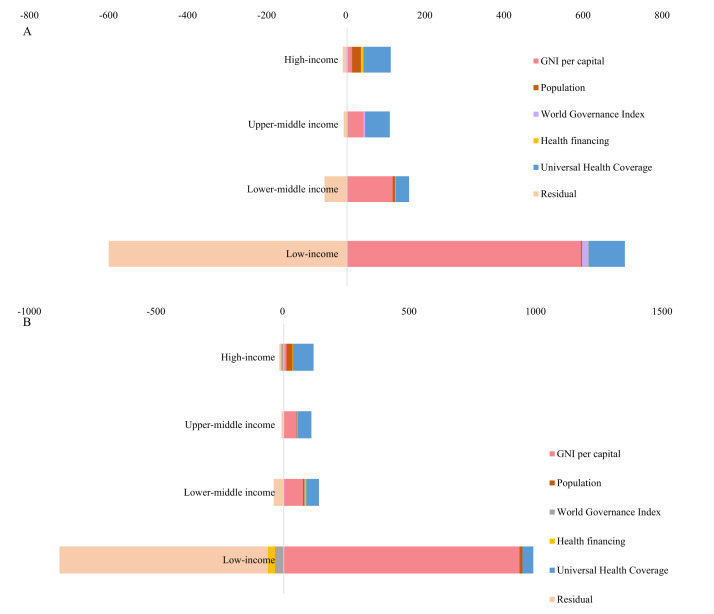
Percentage contribution of economy, social development, and health system in shaping the socioeconomic inequality of health security capacities, by income levels. **Panel A.** In the year 2021. **Panel B**. In the year 2019. GNI – Gross National Income.

We further conducted the decomposition analysis by income levels (**Figure 3**; Table S7 in the **Online Supplementary Document**). The UHC was the leading cause of health security capacity in high-income (69.322%) and upper-middle-income countries (62.461%). For lower-middle (115.700%) and low-income countries (591.679%), GNI per capita was the primary contributor.

### Temporal change of the GHS inequality and decomposition

We also assessed the CI for the GHSI estimation in 2019 and compared it to that in 2021. The average level of the GHSI and its six categories remained essentially unchanged from 2019 to 2021. The CI enlarged from 0.139 in 2019 (*P* < 0.001) to 0.143 in 2021 (*P* < 0.001), however, the difference did not reach statistical significance (Table S4 in the **Online Supplementary Document**). Regarding the six categories of the GHSI, prevention (CI = 0.235 in 2019; CI = 0.248 in 2021), detection and reporting (CI = 0.164 in 2019; CI = 0.167 in 2021), rapid response (CI = 0.106 in 2019; CI = 0.107 in 2021), and risk environment (CI = 0.122 in 2019; CI = 0.126 in 2021) were of widened inequality across countries, with enlarged CI measured from 2019 to 2021. The CI narrowed for the capacity of the health system (CI = 0.228 in 2019; CI = 0.222 in 2021) and compliance with international norms (CI = 0.059 in 2019; CI = 0.057 in 2021); however, the difference did not reach statistical significance.

We also decomposed the changes in the concentration according to the determinants of health security capacity (**Table 1**). According to the first determinant framework, economic status accounted for 70.168%, social development for −62.544%, and the health system accounted for −42.219% of the change of inequality. Under the second determinant framework, the inequality in economic status (679.732%), health financing (266.674%), and health workforce (166.172%) enlarged the global inequality in health security capacity. The UHC development mitigated global inequality by 413.174%, resulting in a reduction in health service delivery inequalities. We also decomposed the changes in the CI of the six GHSI categories (**Figure 4**).

### Sensitivity analysis

We also assessed the country-level core capacity in response to health emergencies through the JEE report, analysing a similar inequality assessment and decomposition using the JEE estimation (Tables S8 and S9 in the **Online Supplementary Document**). There was a similar pro-rich inequality in GHS. When decomposed according to the determinant framework, the health system accounted for 80.384% of the total inequality, among which health service coverage was the leading contributor. We reported this pattern for the four categories in JEE’s capacity evaluation framework (*i.e.* prevention, detection, response, and others).

## DISCUSSION

The COVID-19 pandemic has exposed profound inequalities in country-level capacities for preparedness, detection, and response to health emergencies. Equality and equity have since become a focal point in the negotiations for amendments to the IHR and the Pandemic Agreement. In this study, we quantified disparities in the core capacities outlined by the IHR amendments and assessed the contribution of health system factors in shaping these inequalities. Our findings provide crucial evidence to inform efforts to strengthen GHS and advance equity-oriented global health governance.

Disparities in country-level health security capacities in response to health emergencies have been reported. In this study, high-income countries consistently achieve higher GHSI scores compared to low- and middle-income countries, a trend also reflected in the JEE assessments and its various categories. These disparities in health security capacity persisted and slightly widened between 2019 and 2021, with the CI enlarged from 0.139 to 0.143. The disparity was transformed into public health concerns regarding the performance of diagnostic kits and healthcare service availability during the COVID-19 pandemic, resulting in worsened health outcomes [17,18]. For example, the experience during COVID-19 indicated the GHSI was negatively associated with excess COVID-19 comparative mortality ratios. Greater capacities related to prevention, detection, response, international commitments, and risk environments were each associated with lower comparative mortality ratios [19]. High-income countries dominated the global market in diagnostics, personal protective equipment, therapeutics, and especially vaccines. The WHO and partners designed the Access to COVID-19 Tools Accelerator to accelerate development, production, and equitable access to COVID-19 tests, treatments, and vaccines [20]. As of 3 September 2021, COVAX (the Access to COVID-19 Tools Accelerator’s vaccine pillar) had shipped only 236 million COVID-19 vaccines to 139 countries, leaving most lower-income health workers and vulnerable populations unprotected [21]. By the end of 2021, high-income countries had administered over 70% of the world’s COVID-19 vaccines, while some low- and middle-income countries had vaccinated less than 4% of their populations [5]. Furthermore, prevention, identified as a critical technical area, received the lowest average scores and exhibited the largest disparities across countries. This gap in preventive capacity requires urgent attention. The One Health approach, recognising the interconnection between the health of people, animals, and the environment, has been proposed to improve the ability to detect and prepare for emerging and re-emerging infectious diseases [16].

Health systems, particularly UHC, have been identified as key contributors to socioeconomic inequalities in health security capacities. The analysis revealed that UHC was the leading cause of health security capacity, especially in high-income (69.322%) and upper-middle-income (62.461%) countries. The wave of the West African Ebola epidemic and the COVID-19 pandemic demonstrated the gap between GHS and UHC in global health governance [22]. Strengthening of health systems is a necessary component of epidemic and pandemic preparedness and response, supporting essential public health functions including robust health infrastructure, trained and protected healthcare workers, adequate funding, reliable supply chains, and evidence-based planning and coordination [23]. Effective and accessible primary healthcare can be a key approach for creating cohesion between GHS and UHC [24]. A macro-analysis suggested that countries with stronger UHC frameworks perform better in areas such as disease surveillance, outbreak detection, and emergency response [25]. This improved performance is attributed to broader access to essential healthcare services, a more resilient healthcare workforce, and stronger public health infrastructure. In contrast, nations with fragmented health systems and limited UHC face significant challenges in providing timely healthcare, which exacerbates their vulnerability to health emergencies [26]. Inequalities in healthcare access, financing, and infrastructure lead to diminished health response systems, limiting the ability of disadvantaged populations – particularly lower socioeconomic groups – to benefit from preventive services, diagnostic tools, and timely treatment during emergencies. Addressing these disparities requires the integration of UHC as a core component of GHS frameworks. Strengthening UHC not only improves routine healthcare access but also builds more resilient health systems, enhancing their capacity to respond to emergencies and reducing socioeconomic inequalities in health security [27,28]. However, the WHO framework for strengthening public health capacity, encapsulated in the IHR, was primarily established to foster global commitment to preventing, detecting, and rapidly responding to public health risks and emergencies of international concern. A notable gap in the IHR is its lack of focus on curative services, patient management, and clinical surge capacity during outbreaks. As a result, it does not fully address patient treatment through recovery or the effective containment and resolution of outbreaks and epidemics. Addressing these shortcomings is essential to enhancing both preventive and curative capacities in future health emergencies.

This study identified that from 2019 to 2021, health systems strengthening contributed to a reduction in GHS inequalities, despite the enlarged economic recession. This observation suggested that stronger health security capacity could be achieved through a strengthened health system. The Ebola outbreaks and COVID-19 pandemic necessitated embedding GHS into UHC, creating a single comprehensive health system that meets the IHR requirements for preventing, detecting, and responding to emerging and re-emerging infections, as well as addressing routine health issues. Formulation of a broader, more comprehensive, and thus more secure national health system under UHC would protect and serve the health needs of a population. This national health system must, by design, have several levels of implementation (*i.e.* from community to national administrative levels) and must be multisectoral. Partnerships should understand shared risks and vulnerabilities and ultimately have shared actions to respond to health events early, and be effective in stopping outbreaks, managing patients affected by epidemics, and continuing to offer routine curative services to those in need [29]. Countries with weaker health security often face delayed outbreak detection and response, leading to higher mortality and prolonged disruptions in healthcare systems – effects that disproportionately burden disadvantaged populations and exacerbate existing health and economic inequalities. These vulnerabilities can trigger a cycle of increased out-of-pocket spending, reduced service access, and broader economic losses, reinforcing the disparities identified in our analysis. The findings from this study provide critical evidence to inform global health governance of emergency preparedness and GHS. This study highlights the need for equality-oriented global health governance to strengthen emergency preparedness and health security. The observed socioeconomic disparities in health security capacity underscore the importance of integrating routine healthcare services with emergency preparedness under a unified framework of UHC.

This study has several strengths. We quantified disparities in health security capacity and explored the role of health systems and UHC in shaping socioeconomic inequalities. The findings from this research could guide the development of strategies aimed at achieving a more equitable GHS framework. However, certain limitations must be acknowledged. First, the decomposition of changes in health security inequality was measured between 2019 and 2021; further research with long-term, repeated measurements is needed to confirm the robustness of these findings. Second, we assessed the health security capacity at the country-level; further evaluation regarding the sub-national level was also warranted to inform the health governance within countries.

## CONCLUSIONS

Our results reveal significant disparities in country-level health security capacity and underscore the urgent need for global health governance centred on equality. Strengthening health systems, particularly through the enhancement of the UHC framework by recognising the interconnection between health systems and health security, offers a viable strategy for achieving equality-driven GHS goals.

## Additional material


Online Supplementary Document
