# Supplementary material for: Socioeconomic inequality and decomposition of core capacity in global health security: the role of health system
Source: J Glob Health. 2025 Oct 24;15:04234. doi: 10.7189/jogh.15.04234 (PMC12548770; doi:10.7189/jogh.15.04234)
Supplement: Online Supplementary Document [file jogh-15-04234-s001.pdf]

Supplement to: Wang M, Liu M, Liu Z, Yin H, Xu Z, Ren M. Socioeconomic inequality and decomposition of core capacity in global health security: the role of health system. J Glob Health. 2025;15:04234.

Socioeconomic inequality and decomposition of core capacity in global health security: the role of health system

Supplemental Material

Supplemental Table 1. Detailed information of the determinants of health security capacity and variables applied in this study.

Supplemental Table 2. Association analysis between the socioeconomic determinants and health security capacity.

Supplemental Table 3. Scores for the health security capacities by categories and income levels in 2021.

Supplemental Table 4. Changes in socioeconomic inequalities regarding health security capacities from 2019 to 2021.

Supplemental Table 5. Decomposition of the socioeconomic inequality of country-level health security capacities in response of health emergencies by GHS indicators, applying the decomposition framework 1.

Supplemental Table 6. Decomposition of the socioeconomic inequality of country-

level health security capacities in response of health emergencies by GHS indicators, applying the decomposition framework 2.

Supplemental Table 7. Decomposition of the socioeconomic inequality of country-level health security capacities, by income-levels.

Supplemental Table 8. Decomposition of the socioeconomic inequality of country-level health security capacities in response of health emergencies, by using the JEE estimation, applying the determinant framework 1.

Supplemental Table 9. Decomposition of the socioeconomic inequality of country-level health security capacities in response of health emergencies, by using the JEE estimation, applying the determinant framework 2.

Supplemental Table 1. Detailed information of the determinants of health security capacity and variables applied in this study.

Variable	Definition	Data source
**GNI per capital**	GNI per capita, PPP (current international $)	World Bank Open Data, https://data.worldbank.org/indicator
**Population**	Total population	World Bank Open Data, https://data.worldbank.org/indicator
**World Governance Index**	The Worldwide Governance Indicators (WGI) project constructs aggregate indicators of six broad dimensions of governance: Voice and Accountability; Political Stability and Absence of Violence/Terrorism; Government Effectiveness; Regulatory Quality; Rule of Law; Control of Corruption. The six aggregate indicators are based on over 30 underlying data sources reporting the perceptions of governance of a large number of survey respondents and expert assessments worldwide.	www.govindicators.org
**Health financing**	Domestic general government health expenditure (GGHE-D) as percentage of general government expenditure (GGE) (%)	Global Health Observatory, https://www.who.int/data/gho
**Universal Health Coverage**	UHC Service Coverage Index (SDG 3.8.1)	Global Health Observatory, https://www.who.int/data/gho
**Health workforce**	Medical doctors (per 10,000)	Global Health Observatory, https://www.who.int/data/gho
**Health supplies**	Hospital beds (per 10 000 population)	Global Health Observatory, https://www.who.int/data/gho

Supplemental Table 2. Association analysis between the socioeconomic determinants and health security capacity.

Variable	All countries (n=195)		High-income countries (n=58)		Upper-middle income countries (n=53)		Lower-middle income countries (n=54)		Low-income countries (n=27)	
	β	*P* value	β	*P* value	β	*P* value	β	*P* value	β	*P* value
**Framework 1**										
GNI per capital	0.0002	<0.001	0.0001	0.3120	0.0003	0.1460	0.0004	0.1830	0.0012	0.4110
Population	0.0000	0.0420	0.0000	0.0440	0.0000	0.8260	0.0000	0.0190	0.0000	0.0270
World Governance Index	-2.3527	0.0060	-1.5906	0.5600	-2.5022	0.1370	-0.8868	0.3780	1.8224	0.2250
Health financing	0.3001	0.0870	0.3428	0.3940	-0.0406	0.9010	0.0019	0.9920	0.2311	0.6230
Universal Health Coverage	0.3856	<0.001	1.3041	<0.001	0.7404	<0.001	0.1411	0.1280	0.2643	0.2170
**Framework 2**										
GNI per capital	0.0000	0.5160	-0.0001	0.5660	-	-	0.0006	0.5850	-	-
Population	0.0000	0.0010	0.0000	0.1380	-	-	0.0000	0.0420	-	-
World Governance Index	3.4410	0.0750	4.2603	0.2890	-	-	3.6494	0.4080	-	-
Health financing	-0.1131	0.7260	-0.3782	0.6070	-	-	-0.6986	0.3960	-	-
Universal Health Coverage	0.4862	0.0360	1.4382	0.1180	-	-	0.2918	0.4130	-	-
Health workforce	0.0120	0.9480	-0.0858	0.8120	-	-	0.3797	0.5400	-	-
Health supplies	0.0308	0.5770	-0.0126	0.9080	-	-	-0.1024	0.5920	-	-

Supplemental Table 3. Scores for the health security capacities by categories and income levels in 2021.

	All countries (n=195)	High-income countries (n=58)	Upper-middle income countries (n=53)	Lower-middle income countries (n=54)	Low-income countries (n=27)
**Total Score**					
Average score (95% CI)	38.91 (36.98-40.83)	50.69 (47.17-54.21)	39.86 (36.57-43.14)	32.54 (30.85-34.22)	26.40 (23.88-28.92)
0-20 (number, proportion)	8 (4.10)	1 (1.72)	2 (3.77)	0 (0.00)	5 (18.52)
20-40 (number, proportion)	110 (56.41)	14 (24.14)	24 (45.28)	47 (87.04)	22 (81.48)
40-60 (number, proportion)	58 (29.74)	26 (44.83)	25 (47.17)	7 (12.96)	0 (0.00)
60-80 (number, proportion)	19 (9.74)	17 (29.31)	2 (3.77)	0 (0.00)	0 (0.00)
80-100 (number, proportion)	0 (0.00)	0 (0.00)	0 (0.00)	0 (0.00)	0 (0.00)
**Prevention**					
Average score (95% CI)	28.45 (25.93-30.97)	42.22 (37.72-46.72)	30.40 (25.45-35.36)	19.96 (17.12-22.81)	13.97 (11.83-16.11)
0-20 (number, proportion)	79 (4.93)	8 (13.79)	16 (31.37)	28 (51.85)	24 (88.89)
20-40 (number, proportion)	57 (29.53)	14 (24.14)	17 (33.33)	23 (42.59)	3 (11.11)
40-60 (number, proportion)	48 (24.87)	29 (50.00)	16 (31.37)	3 (5.56)	0 (0.00)
60-80 (number, proportion)	9 (4.66)	7 (12.07)	2 (3.92)	0 (0.00)	0 (0.00)
80-100 (number, proportion)	0 (0.00)	0 (0.00)	0 (0.00)	0 (0.00)	0 (0.00)
**Detection and reporting**					
Average score (95% CI)	32.26 (29.46-35.06)	42.85 (37.00-48.71)	33.84 (28.39-39.28)	26.58 (22.73-30.43)	21.03 (16.94-25.13)
0-20 (number, proportion)	53 (27.89)	11 (19.30)	10 (19.61)	19 (35.19)	11 (42.31)
20-40 (number, proportion)	79 (41.58)	14 (24.56)	24 (47.06)	26 (48.15)	15 (57.69)
40-60 (number, proportion)	37 (19.47)	16 (28.07)	12 (23.53)	9 (16.67)	0 (0.00)
60-80 (number, proportion)	18 (9.47)	14 (24.56)	4 (7.84)	0 (0.00)	0 (0.00)

80-100 (number, proportion)	3 (1.58)	2 (3.51)	1 (1.96)	0 (0.00)	0 (0.00)
**Rapid response**					
Average score (95% CI)	37.58 (35.88-39.28)	46.35 (43.38-49.31)	39.31 (36.13-42.48)	32.18 (30.20-34.16)	27.11 (23.87-30.34)
0-20 (number, proportion)	6 (3.08)	0 (0.00)	0 (0.00)	2 (3.70)	4 (3.13)
20-40 (number, proportion)	122 (62.56)	20 (34.48)	34 (64.15)	44 (81.48)	119 (61.98)
40-60 (number, proportion)	55 (28.21)	31 (53.45)	14 (26.42)	8 (14.81)	55 (28.65)
60-80 (number, proportion)	12 (6.15)	7 (12.07)	5 (9.43)	0 (0.00)	12 (6.25)
80-100 (number, proportion)	0 (0.00)	0 (0.00)	0 (0.00)	0 (0.00)	0 (0.00)
**Health system**					
Average score (95% CI)	31.49 (28.86-34.12)	45.56 (40.82-50.30)	33.83 (28.81-38.86)	22.58 (19.52-25.63)	16.66 (13.49-19.83)
0-20 (number, proportion)	73 (37.44)	9 (15.52)	17 (32.08)	26 (48.15)	18 (66.67)
20-40 (number, proportion)	54 (27.69)	8 (13.79)	15 (28.30)	22 (40.74)	9 (33.33)
40-60 (number, proportion)	51 (26.15)	28 (48.28)	17 (32.08)	6 (11.11)	0 (0.00)
60-80 (number, proportion)	17 (8.72)	13 (22.41)	4 (7.55)	0 (0.00)	0 (0.00)
80-100 (number, proportion)	0 (0.00)	0 (0.00)	0 (0.00)	0 (0.00)	0 (0.00)
**Compliance with international norms**					
Average score (95% CI)	47.82 (45.91-49.72)	54.62 (50.46-58.78)	46.76 (43.68-49.84)	44.77 (41.92-47.63)	43.91 (39.33-48.50)
0-20 (number, proportion)	3 (1.54)	3 (5.17)	0 (0.00)	0 (0.00)	0 (0.00)
20-40 (number, proportion)	55 (28.21)	6 (10.34)	17 (32.08)	18 (33.33)	11 (40.74)
40-60 (number, proportion)	97 (49.74)	25 (43.10)	30 (56.60)	29 (53.70)	13 (48.15)
60-80 (number, proportion)	39 (20.00)	23 (39.66)	6 (11.32)	7 (12.96)	3 (11.11)
80-100 (number, proportion)	1 (0.51)	1 (1.72)	0 (0.00)	0 (0.00)	0 (0.00)
**Risk environment**					
Average score (95% CI)	55.84 (53.74-57.94)	72.52 (70.15-74.90)	55.00 (52.75-57.25)	49.12 (46.99-51.25)	35.75 (32.63-38.86)
0-20 (number, proportion)	29 (14.87)	0 (0.00)	3 (5.66)	7 (12.96)	18 (66.67)

20-40 (number, proportion)	92 (47.18)	5 (8.62)	36 (67.92)	40 (74.07)	9 (33.33)
40-60 (number, proportion)	62 (31.79)	41 (70.69)	14 (26.42)	7 (12.96)	0 (0.00)
60-80 (number, proportion)	12 (6.15)	12 (20.69)	0 (0.00)	0 (0.00)	0 (0.00)
80-100 (number, proportion)	0 (0.00)	0 (0.00)	0 (0.00)	0 (0.00)	0 (0.00)

Supplemental Table 4. Changes in socioeconomic inequalities regarding health security capacities from 2019 to 2021.

	Concentration index in 2019	Concentration index in 2021	Changes of Concentration index	*P* Value
**All countries**				
Total Score	0.1394	0.1426	0.0031	0.8267
Prevention	0.2351	0.2477	0.0126	0.6346
Detection and reporting	0.1642	0.1668	0.0026	0.9330
Rapid response	0.1061	0.1069	0.0008	0.9554
Health system	0.2276	0.2215	-0.0061	0.8206
Compliance with international norms	0.0587	0.0573	-0.0014	0.9278
Risk environment	0.1223	0.1263	0.0040	0.6191
**High-income countries**				
Total Score	0.0722	0.0698	-0.0024	0.9281
Prevention	0.1012	0.1015	0.0003	0.9944
Detection and reporting	0.1222	0.1115	-0.0107	0.8385
Rapid response	0.0661	0.0574	-0.0087	0.7369
Health system	0.0970	0.0907	-0.0063	0.8795
Compliance with international norms	0.0317	0.0348	0.0030	0.9163
Risk environment	0.0470	0.0474	0.0003	0.9730
**Upper-middle income countries**				
Total Score	0.0935	0.0934	-0.0001	0.9984
Prevention	0.1799	0.1840	0.0041	0.9425
Detection and reporting	0.1349	0.1341	-0.0008	0.9902

Rapid response	0.0594	0.0432	-0.0162	0.6205
Health system	0.1631	0.1505	-0.0127	0.8197
Compliance with international norms	0.0711	0.0751	0.0040	0.8680
Risk environment	0.0282	0.0341	0.0059	0.6926
**Lower-middle income countries**				
Total Score	0.0463	0.0504	0.0041	0.8347
Prevention	0.1439	0.1654	0.0214	0.6639
Detection and reporting	0.0611	0.0873	0.0263	0.6501
Rapid response	0.0306	0.0005	-0.0301	0.2257
Health system	0.1017	0.1090	0.0074	0.8900
Compliance with international norms	-0.0153	-0.0089	0.0064	0.8047
Risk environment	0.0412	0.0433	0.0021	0.8893
**Low-income countries**				
Total Score	0.0243	0.0225	-0.0018	0.9585
Prevention	0.0666	0.0507	-0.0159	0.7860
Detection and reporting	0.0143	0.0356	0.0213	0.7334
Rapid response	0.0462	0.0424	-0.0038	0.9189
Health system	0.0040	-0.0037	-0.0078	0.9221
Compliance with international norms	-0.0028	-0.0006	0.0022	0.9627
Risk environment	0.0343	0.0279	-0.0064	0.8527

Supplemental Table 5. Decomposition of the socioeconomic inequality of country-level health security capacities in response of health emergencies by GHS indicators, applying the decomposition framework 1.

	Year of 2019					Year of 2021					Contribution to the change	
**Total Score**	Elasticity	Concentration index	Contribution	Percentage contribution (%)		Elasticity	Concentration index	Contribution	Percentage contribution (%)		Contribution	Percentage contribution to the change (%)
GNI per capital	0.1338	0.5026	0.0673	49.3368		0.1259	0.5055	0.0636	44.6386		0.0044	70.1680
Population	0.0100	-0.0443	-0.0004	-0.3241		0.0090	-0.0227	-0.0002	-0.1433		0.0002	2.7619
World Governance Index	-0.1755	0.1254	-0.0220	-16.1407		-0.1471	0.1283	-0.0189	-13.2425		-0.0041	-65.3056
Health financing	0.0730	0.1724	0.0126	9.2306		0.0865	0.1724	0.0149	10.4625		-0.0023	-37.4490
Universal Health Coverage	0.6462	0.1260	0.0814	59.7394		0.6466	0.1257	0.0813	56.9911		-0.0003	-4.7698
Residual			-0.0025	-1.8420				0.0018	1.2937			
**Prevention**												
GNI per capital	0.1900	0.5026	0.0955	42.0829		0.1887	0.5055	0.0954	38.5027		0.0012	5.9191
Population	0.0108	-0.0443	-0.0005	-0.2102		0.0122	-0.0227	-0.0003	-0.1121		0.0003	1.4323
World Governance Index	-0.4487	0.1254	-0.0563	-24.7950		-0.4198	0.1283	-0.0539	-21.7479		-0.0050	-23.8395

Health financing	0.0853	0.1724	0.0147	6.4839		0.2204	0.1724	0.0380	15.3410		-0.0233	-112.0994
Universal Health Coverage	1.4261	0.1260	0.1797	79.2207		1.3280	0.1257	0.1669	67.3815		0.0118	56.8956
Residual			-0.0063	-2.7824				0.0016	0.6349			
**Detection and reporting**												
GNI per capital	0.2269	0.5026	0.1140	71.4301		0.1980	0.5055	0.1001	60.0207		0.0152	212.4848
Population	0.0215	-0.0443	-0.0010	-0.5967		0.0207	-0.0227	-0.0005	-0.2810		0.0004	5.9734
World Governance Index	-0.5656	0.1254	-0.0709	-44.4165		-0.5197	0.1283	-0.0667	-39.9793		-0.0074	-104.0140
Health financing	0.1885	0.1724	0.0325	20.3624		0.1590	0.1724	0.0274	16.4384		0.0051	71.1605
Universal Health Coverage	0.7209	0.1260	0.0909	56.9176		0.8361	0.1257	0.1051	63.0002		-0.0148	-207.2398
Residual			-0.0059	-3.6968				0.0013	0.8009			
**Rapid response**												
GNI per capital	0.1157	0.5026	0.0582	55.0112		0.1046	0.5055	0.0529	49.4710		0.0059	496.5285
Population	0.0069	-0.0443	-0.0003	-0.2902		0.0031	-0.0227	-0.0001	-0.0657		0.0000	-1.6982
World Governance Index	-0.1549	0.1254	-0.0194	-18.3728		-0.0317	0.1283	-0.0041	-3.8087		-0.0159	-1335.5146
Health financing	0.0500	0.1724	0.0086	8.1514		0.0983	0.1724	0.0169	15.8474		-0.0083	-699.5284
Universal Health Coverage	0.4863	0.1260	0.0613	57.9799		0.3226	0.1257	0.0405	37.9167		0.0205	1717.8285
Residual			-0.0026	-2.4794				0.0007	0.6392			
**Health system**												

GNI per capital	0.1895	0.5026	0.0952	43.3475		0.1686	0.5055	0.0852	38.4742		0.0110	582.0883
Population	0.0236	-0.0443	-0.0010	-0.4768		0.0193	-0.0227	-0.0004	-0.1975		0.0003	16.7572
World Governance Index	-0.4019	0.1254	-0.0504	-22.9412		-0.4111	0.1283	-0.0527	-23.8088		0.0000	-2.0308
Health financing	0.2057	0.1724	0.0355	16.1528		0.1925	0.1724	0.0332	14.9847		0.0023	119.9166
Universal Health Coverage	1.1508	0.1260	0.1450	66.0379		1.2114	0.1257	0.1522	68.7157		-0.0081	-426.4285
Residual			-0.0047	-2.1202				0.0041	1.8318			
**Compliance with international norms**												
GNI per capital	0.0606	0.5026	0.0305	53.4687		0.0707	0.5055	0.0358	62.3731		-0.0049	-1404.3096
Population	0.0013	-0.0443	-0.0001	-0.0974		0.0008	-0.0227	0.0000	-0.0309		0.0000	1.7633
World Governance Index	-0.0965	0.1254	-0.0121	-21.2390		-0.1326	0.1283	-0.0170	-29.6813		0.0042	1212.9884
Health financing	-0.0260	0.1724	-0.0045	-7.8637		-0.0100	0.1724	-0.0017	-3.0119		-0.0028	-787.7470
Universal Health Coverage	0.3542	0.1260	0.0446	78.3734		0.3147	0.1257	0.0396	69.0041		0.0048	1386.3261
Residual			-0.0015	-2.6421				0.0008	1.3470			
**Risk environment**												
GNI per capital	0.1019	0.5026	0.0512	41.9501		0.0900	0.5055	0.0455	36.0045		0.0063	146.3480
Population	0.0060	-0.0443	-0.0003	-0.2163		0.0059	-0.0227	-0.0001	-0.1060		0.0001	2.9437
World Governance Index	0.2085	0.1254	0.0261	21.4239		0.2645	0.1283	0.0339	26.8727		-0.0064	-149.4848

Health financing	0.0371	0.1724	0.0064	5.2392		-0.0095	0.1724	-0.0016	-1.2914		0.0080	187.2003
Universal Health Coverage	0.3014	0.1260	0.0380	31.1309		0.3732	0.1257	0.0469	37.1308		-0.0092	-213.8910
Residual			0.0006	0.4721				0.0018	1.3895			

Supplemental Table 6. Decomposition of the socioeconomic inequality of country-level health security capacities in response of health emergencies by GHS indicators, applying the decomposition framework 2.

	**Year of 2019**					**Year of 2021**					**Contribution to the change**	
**Total Score**	**Elasticity**	**Concentration index**	**Contribution**	**Percentage contribution (%)**		**Elasticity**	**Concentration index**	**Contribution**	**Percentage contribution (%)**		**Contribution**	**Percentage contribution to the change (%)**
Health financing	0.064	0.172	0.011	8.084		-0.033	0.172	-0.006	-3.943		0.017	266.674
Universal Health Coverage	0.594	0.126	0.075	54.904		0.815	0.126	0.102	71.866		-0.028	-450.970
Health workforce	0.037	0.337	0.013	9.258		0.007	0.343	0.003	1.768		0.010	166.172
Health supplies	0.009	0.191	0.002	1.317		0.023	0.169	0.004	2.734		-0.003	-45.152
Population	0.009	-0.044	0.000	-0.291		0.083	-0.023	-0.002	-1.327		0.003	55.902
World Governance Index	0.009	0.125	0.001	0.857		0.215	0.128	0.028	19.368		-0.026	-413.174

GNI per capital	0.113	0.503	0.057	41.574		0.029	0.505	0.015	10.293		0.042	679.732
Residual			-0.021	-15.703				-0.001	-0.759			
**Prevention**												
Health financing	0.083	0.172	0.014	6.333		-0.010	0.172	-0.002	-0.688		0.016	77.341
Universal Health Coverage	1.169	0.126	0.147	64.932		1.214	0.126	0.153	61.593		-0.006	-29.469
Health workforce	0.192	0.337	0.065	28.546		0.188	0.343	0.064	25.989		0.003	12.882
Health supplies	-0.028	0.191	-0.005	-2.363		0.029	0.169	0.005	1.975		-0.010	-49.444
Population	0.012	-0.044	-0.001	-0.243		0.101	-0.023	-0.002	-0.925		0.004	20.160
World Governanc e Index	-0.104	0.125	-0.013	-5.731		0.144	0.128	0.018	7.459		-0.031	-150.914
GNI per capital	0.120	0.503	0.060	26.659		0.036	0.505	0.018	7.254		0.043	206.841
Residual			-0.041	-18.133				-0.007	-2.657			
**Detection and reporting**												
Health financing	0.203	0.172	0.035	21.977		0.090	0.172	0.016	9.330		0.020	273.338

Universal Health Coverage	0.546	0.126	0.069	43.142		1.411	0.126	0.177	106.307		-0.109	-1529.018
Health workforce	0.040	0.337	0.013	8.356		-0.245	0.343	-0.084	-50.415		0.096	1347.283
Health supplies	0.010	0.191	0.002	1.199		0.028	0.169	0.005	2.868		-0.004	-52.026
Population	0.023	-0.044	-0.001	-0.649		0.158	-0.023	-0.004	-2.143		0.006	90.256
World Governance Index	-0.139	0.125	-0.017	-10.934		0.186	0.128	0.024	14.285		-0.041	-576.266
GNI per capital	0.179	0.503	0.090	56.214		0.086	0.505	0.044	26.176		0.047	656.309
Residual			-0.031	-19.305				-0.011	-6.409			
**Rapid response**												
Health financing	-0.001	0.172	0.000	-0.182		-0.144	0.172	-0.025	-23.172		0.025	2064.773
Universal Health Coverage	0.842	0.126	0.106	100.389		1.113	0.126	0.140	130.809		-0.034	-2893.813
Health workforce	-0.066	0.337	-0.022	-21.164		-0.069	0.343	-0.024	-22.214		0.001	47.268
Health supplies	-0.005	0.191	-0.001	-0.836		0.002	0.169	0.000	0.394		-0.001	-105.650
Population	0.005	-0.044	0.000	-0.228		0.102	-0.023	-0.002	-2.173		0.004	370.481

World Governance Index	-0.009	0.125	-0.001	-1.018		0.326	0.128	0.042	39.177		-0.042	-3529.386
GNI per capital	0.091	0.503	0.046	43.221		0.025	0.505	0.013	11.794		0.033	2807.014
Residual			-0.021	-20.182				-0.037	-34.614			
**Health system**												
Health financing	0.208	0.172	0.036	16.334		-0.012	0.172	-0.002	-0.965		0.038	2007.268
Universal Health Coverage	1.029	0.126	0.130	59.049		1.627	0.126	0.204	92.270		-0.076	-3998.857
Health workforce	0.027	0.337	0.009	4.131		-0.059	0.343	-0.020	-9.053		0.029	1528.397
Health supplies	0.098	0.191	0.019	8.516		0.047	0.169	0.008	3.574		0.008	401.271
Population	0.020	-0.044	-0.001	-0.404		0.099	-0.023	-0.002	-1.018		0.004	208.412
World Governance Index	-0.089	0.125	-0.011	-5.093		-0.003	0.128	0.000	-0.179		-0.011	-584.192
GNI per capital	0.115	0.503	0.058	26.328		-0.009	0.505	-0.005	-2.072		0.063	3312.797
Residual			-0.019	-8.860				0.039	17.443			
**Compliance with**												

**international norms**												
Health financing	-0.003	0.172	0.000	-0.841		-0.086	0.172	-0.015	-25.798		0.014	4095.864
Universal Health Coverage	0.152	0.126	0.019	33.584		0.138	0.126	0.017	30.260		0.002	480.357
Health workforce	0.091	0.337	0.031	53.910		0.061	0.343	0.021	36.195		0.011	3112.200
Health supplies	0.010	0.191	0.002	3.459		0.047	0.169	0.008	13.711		-0.007	-2043.613
Population	0.000	-0.044	0.000	-0.024		0.077	-0.023	-0.002	-3.055		0.003	976.224
World Governance Index	-0.020	0.125	-0.003	-4.435		0.235	0.128	0.030	52.508		-0.032	-9156.118
GNI per capital	0.081	0.503	0.041	71.500		-0.011	0.505	-0.006	-9.666		0.046	13302.327
Residual			-0.033	-57.155				0.003	5.847			
**Risk environment**												
Health financing	0.010	0.172	0.002	1.386		-0.005	0.172	-0.001	-0.679		0.003	59.452
Universal Health Coverage	0.280	0.126	0.035	28.882		0.184	0.126	0.023	18.317		0.012	278.386

Health workforce	-0.006	0.337	-0.002	-1.739		0.104	0.343	0.036	28.154		-0.037	-865.596
Health supplies	-0.009	0.191	-0.002	-1.365		-0.001	0.169	0.000	-0.122		-0.001	-30.288
Population	0.004	-0.044	0.000	-0.135		0.016	-0.023	0.000	-0.281		0.001	14.204
World Governance Index	0.236	0.125	0.030	24.259		0.300	0.128	0.039	30.507		-0.007	-171.495
GNI per capital	0.116	0.503	0.058	47.696		0.052	0.505	0.026	20.845		0.032	755.062
Residual			0.001	1.016				0.004	3.260			

Supplemental Table 7. Decomposition of the socioeconomic inequality of country-level health security capacities, by income-levels.

	Year of 2019					Year of 2021					Contribution to the change	
**High-income**	**Elasticity**	**Concentration index**	**Contribution**	**Percentage contribution (%)**		**Elasticity**	**Concentration index**	**Contribution**	**Percentage contribution (%)**		**Contribution**	**Percentage contribution to the change (%)**
GNI per capital	0.032	0.213	0.007	11.312		0.039	0.230	0.009	12.848		-0.001	-9.790
Population	0.056	0.255	0.014	23.231		0.060	0.255	0.015	22.084		-0.001	-12.988
World Governance Index	-0.264	0.021	-0.006	-9.281		-0.099	0.016	-0.002	-2.305		-0.002	-24.553
Health financing	0.024	0.043	0.001	1.688		0.099	0.047	0.005	6.591		-0.003	-36.580
Universal Health Coverage	2.688	0.019	0.050	81.490		2.187	0.022	0.048	69.322		0.019	217.551
Residual			-0.005	-8.439				-0.006	-8.540			
**Upper-middle income**												
GNI per capital	0.231	0.197	0.045	49.612		0.179	0.215	0.039	41.219		0.015	749.396

Population	0.003	0.177	0.001	0.623		-0.001	0.293	0.000	-0.463		0.001	61.910
World Governance Index	-0.194	-0.030	0.006	6.259		-0.156	-0.028	0.004	4.684		0.001	40.436
Health financing	0.001	0.035	0.000	0.048		-0.012	0.057	-0.001	-0.708		0.000	24.669
Universal Health Coverage	1.163	0.041	0.048	52.564		1.242	0.047	0.058	62.461		0.003	170.457
Residual			-0.008	-9.106				-0.007	-7.193			
**Lower-middle income**												
GNI per capital	0.130	0.270	0.035	75.705		0.211	0.276	0.058	115.697		-0.021	-513.231
Population	0.010	0.222	0.002	4.854		0.010	0.268	0.003	5.281		0.001	12.405
World Governance Index	-0.089	-0.015	0.001	2.842		-0.055	-0.017	0.001	1.824		0.001	15.607
Health financing	0.022	0.110	0.002	5.313		0.001	0.152	0.000	0.164		0.003	80.818

Universal Health Coverage	0.304	0.077	0.023	50.786		0.237	0.072	0.017	33.949		0.004	91.015
Residual			-0.018	-39.500				-0.029	-56.915			
**Low-income**												
GNI per capital	1.050	0.199	0.209	931.875		0.731	0.182	0.133	591.679		0.046	118926.072
Population	0.090	0.028	0.003	11.362		0.093	0.006	0.001	2.468		-0.002	-5467.613
World Governance Index	0.146	-0.053	-0.008	-34.734		0.114	0.032	0.004	16.150		0.011	27914.194
Health financing	0.091	-0.068	-0.006	-27.643		0.067	0.003	0.000	0.786		0.005	12436.729
Universal Health Coverage	0.210	0.046	0.010	43.050		0.443	0.046	0.021	91.540		-0.011	-27759.605
Residual			-0.185	-823.910				-0.136	-602.622			

Supplemental Table 8. Decomposition of the socioeconomic inequality of country-level health security capacities in response of health emergencies, by using the JEE estimation, applying the determinant framework 1.

	Elasticity	Concentration index	Contribution	Percentage contribution (%)
**Total Score**				
GNI per capital	0.112	0.503	0.056	35.181
Population	0.038	-0.044	-0.002	-1.064
World Governance Index	-0.016	0.125	-0.002	-1.257
Health financing	-0.090	0.172	-0.016	-9.720
Universal Health Coverage	1.061	0.126	0.134	83.650
Residual			-0.011	-6.790
**Prevention**				
GNI per capital	0.099	0.503	0.050	32.815
Population	0.039	-0.044	-0.002	-1.140
World Governance Index	0.032	0.125	0.004	2.628
Health financing	-0.046	0.172	-0.008	-5.184
Universal Health Coverage	0.947	0.126	0.119	78.823
Residual			-0.012	-7.942
**Detection**				
GNI per capital	0.077	0.503	0.039	33.510
Population	0.038	-0.044	-0.002	-1.456
World Governance Index	0.027	0.125	0.003	2.937
Health financing	-0.038	0.172	-0.007	-5.655
Universal Health Coverage	0.658	0.126	0.083	71.903

Residual			-0.001	-1.238
**Response**				
GNI per capital	0.156	0.503	0.079	45.106
Population	0.037	-0.044	-0.002	-0.953
World Governance Index	-0.045	0.125	-0.006	-3.243
Health financing	-0.181	0.172	-0.031	-17.906
Universal Health Coverage	1.169	0.126	0.147	84.576
Residual			-0.013	-7.581
**Others**				
GNI per capital	0.128	0.503	0.065	28.829
Population	0.055	-0.044	-0.002	-1.081
World Governance Index	-0.054	0.125	-0.007	-3.018
Health financing	-0.027	0.172	-0.005	-2.078
Universal Health Coverage	1.408	0.126	0.177	79.319
Residual			-0.004	-1.971

Supplemental Table 9. Decomposition of the socioeconomic inequality of country-level health security capacities in response of health emergencies, by using the JEE estimation, applying the determinant framework 2.

	Elasticity	Concentration index	Contribution	Percentage contribution (%)
**Total score**				
Health financing	-0.353	0.172	-0.061	-38.052
Universal Health Coverage	1.477	0.126	0.186	116.411
Health workforce	0.018	0.337	0.006	3.732
Health supplies	-0.014	0.191	-0.003	-1.707
Population	0.046	-0.044	-0.002	-1.261
World Governance Index	0.086	0.125	0.011	6.728
GNI per capital	0.131	0.503	0.066	41.302
Residual			-0.043	-27.153
**Prevention**				
Health financing	-0.273	0.172	-0.047	-31.059
Universal Health Coverage	1.074	0.126	0.135	89.441
Health workforce	0.008	0.337	0.003	1.839
Health supplies	0.029	0.191	0.006	3.637
Population	0.039	-0.044	-0.002	-1.156

World Governance Index	0.158	0.125	0.020	13.069
GNI per capital	0.152	0.503	0.076	50.373
Residual			-0.040	-26.146
**Detection**				
Health financing	-0.095	0.172	-0.016	-14.158
Universal Health Coverage	0.470	0.126	0.059	51.440
Health workforce	0.058	0.337	0.019	16.824
Health supplies	0.005	0.191	0.001	0.854
Population	0.029	-0.044	-0.001	-1.117
World Governance Index	0.083	0.125	0.010	9.027
GNI per capital	0.101	0.503	0.051	43.914
Residual			-0.008	-6.784
**Response**				
Health financing	-0.440	0.172	-0.076	-43.563
Universal Health Coverage	1.639	0.126	0.207	118.599
Health workforce	0.019	0.337	0.006	3.655
Health supplies	-0.071	0.191	-0.013	-7.724
Population	0.041	-0.044	-0.002	-1.047
World Governance Index	0.024	0.125	0.003	1.745
GNI per capital	0.179	0.503	0.090	51.742

Residual			-0.041	-23.407
**Others**				
Health financing	-0.348	0.172	-0.060	-26.855
Universal Health Coverage	1.948	0.126	0.246	109.744
Health workforce	0.006	0.337	0.002	0.938
Health supplies	0.002	0.191	0.000	0.205
Population	0.048	-0.044	-0.002	-0.940
World Governance Index	-0.067	0.125	-0.008	-3.772
GNI per capital	0.152	0.503	0.076	34.154
Residual			-0.030	-13.474
